# Scorpion Sting: A Hurt to the Heart Reported in a Tertiary Care Hospital in Central Rural India

**DOI:** 10.7759/cureus.32536

**Published:** 2022-12-14

**Authors:** Shashank Banait, Krupali Thakre, Tanvi Banait, Jyoti Jain, Manish Patode

**Affiliations:** 1 Ophthalmology, Jawaharlal Nehru Medical College, Datta Meghe Institute of Medical Sciences (Deemed to be University), Wardha, IND; 2 Medicine, Mahatma Gandhi Institute of Medical Sciences, Wardha, IND; 3 Medicine, Jawaharlal Nehru Medical College, Datta Meghe Institute of Medical Sciences (Deemed to be University), Wardha, IND; 4 Department of Medicine, Mahatma Gandhi Institute of Medical Sciences, Wardha, IND

**Keywords:** prazosin, acute myocardial injury, echocardiogram, electrocardiographic changes, scorpion sting

## Abstract

Scorpion stings are painful but harmless and are rarely life-threatening. There is emerging evidence of the association of electrocardiographic (ECG) changes in patients following scorpion stings. We report a case of scorpion sting in a patient in central rural India and provide a review of similar published cases. A 55-year-old previously healthy female was hospitalized in the department of medicine at our institute within two hours of a scorpion sting. She presented with severe pain at the site of the sting and profuse sweating. Her routine investigations (complete blood count renal function test, liver function tests, and arterial blood gas analysis) results were normal. Her electrocardiogram revealed acute myocardial infarction, and serial ECG showed ST and T-wave changes. On laboratory investigation, it was found that her troponin-T was positive and creatinine phosphokinase levels were raised. There was apical wall hypokinesia on transthoracic echocardiography on Day 1 and Day 2. The patient recovered completely and was discharged within five days of hospitalization once her symptoms improved. This case highlights the severe presentation of scorpion stings in otherwise healthy females. The chances of improved clinical symptoms are more if prazosin (125-250 ug) is administered early after scorpion-stung patients experience cardiac abnormalities. This treatment can dramatically alter scorpion envenomation’s morbidity and mortality depending on the duration after which it is administered. This case raised our interest due to cardiovascular manifestations in the patient and the early treatment with prazosin for the scorpion sting. Hence, this case was reported for the purpose of creating awareness among physicians and protecting the more vulnerable population.

## Introduction

Around 86 species of scorpions are found in India; among them, stings of two species, namely, *Mesobuthus tamulus *(Indian red scorpion) and *Heterometrus swammerdami*, are known to cause serious complications. Indian red scorpion sting mainly manifests as cardiovascular symptoms [1] Scorpion venom stimulates the sympathetic and parasympathetic postganglionic nervous system through voltage-dependent ion channels. In most cases, stimulation of the sympathetic nervous system predominates, which leads to a “sympathetic storm.” Most scorpion stings are benign and patients present with local pain, tachycardia, systemic hypertension, and cold extremities. However, some patients may present with fatal severe complications such as acute pulmonary edema, acute myocardial stunning, acute heart failure, and acute respiratory distress syndrome due to the stimulation of alpha receptors and direct stimulant actions on the heart by scorpion toxins. These autonomic disturbances result from the unopposed effects of postsynaptic alpha-receptor stimulation. This is the rationale for treatment with alpha-blocker prazosin, as it blocks alpha-receptor activity [2,3]. There are many reports of cardiovascular system involvement in children who had a scorpion sting. Reports of cardiovascular system involvement after scorpion stings among adults are sparse, and therefore, many questions are unanswered about the pathophysiology, disease characteristics, and management of scorpion stings.

## Case presentation

A 55-year-old female, with no past medical history, presented with a scorpion sting on her right thumb to our emergency department. She had complaints of severe pain at the sting site and profuse sweating within two hours of the sting. There was no history of chest pain, back pain, palpitations, vomiting, syncope, or breathlessness. She was conscious, pale, diaphoretic, and afebrile on general examination. She had a heart rate of 82 beats /minute, blood pressure (BP) of 122/84 mm of Hg, respiratory rate of 24 breaths/minute, and oxygen saturation (SPO2) of 98% on room air. Other findings of the general examination were normal. An examination of the right thumb (the sting site) revealed local swelling with redness. The systemic examination did not reveal any abnormality on admission. Table 1 shows the patient’s vital signs on admission.

**Table 1:** Patient vital signs on admission and during the hospital stay.

Vital signs	On admission (Day 1)	Day 2	Day 3	Normal values
Temperature	37.1 °C	37.5 °C	37 °C	36.1-37.2 °C
Heart rate	82/minute	78-92/ minute	76-88/ minute	60-100/ minute
Respiratory rate	24/ minute	20/ minute	18/ minute	12-20/ minute
Blood pressure	122/84 mmHg	130/88 mmHg	120/76 mmHg	140/90 mmHg
Oxygen saturation	98%	98%	98%	>94%

Blood work was significant for hemoglobin at 9.7 gm/dl (normocytic to microcytic mildly hypochromic red blood corpuscles) and also revealed positive troponin T, metabolic acidosis, and positive creatinine phosphokinase of 493 mg/dl. The patient’s arterial blood gas analysis is suggestive of uncomplicated high anion gap metabolic acidosis with compensatory respiratory alkalosis. Table 2 shows the laboratory values during the hospital course.

**Table 2:** The patient’s laboratory values on admission.

Laboratory values	On admission	Reference values
Complete blood count		
Hemoglobin (gm/dL)	9.7	11-14 gm/dl
Mean corpuscular volume (MCV) (femtolitre)	75	80-100 femtolitre
Mean corpuscular hemoglobin (MCH) (picogram)	25.7	27-31 picogram
Mean corpuscular hemoglobin concentration (MCHC) ( gm/dl)	34.3	33-37 gm/dl
Red cell distribution width (RDW) (%)	16.5	11.6-13.7 %
White blood cells (in K/uL)	10.56	4-11
Neutrophils (%)	92	60-70 %
Lymphocytes (%)	6	20-30 %
Platelets (in lacs/uL)	228	150-350
Renal function test		
Serum creatinine	1.10	0.72-1.18 mg/dl
Serum urea	40	17-43 mg/dl
Serum sodium	141	136-146 mEq/L
Serum potassium	3.98	3.5-5.1 mEq/L
Liver function tests		
Serum protein (gm/dl)	6.91	6.6-8.3 gm/dl
Serum albumin (gm/dl)	3.88	3.5-5.2 gm/dl
Total Bilirubin (mg/dl)	0.37	0.3-1.2 mg/dl
Alanine transaminase (IU/L)	12	<50 IU/L
Aspartate transaminase (IU/L)	25	<50 IU/L
Alkaline phosphatase (IU/L)	60	80-300 IU/L
Arterial blood gas analysis		
pH	7.31	7.35-7.45
PaCO2	31.2	35-45 mmHg
PaO2	40	75-100 mm Hg
HCO3	18.4	23-29 mEq/L
Anion gap	18	4-12 mEq/L
Cardiac biomarkers		
Troponin-T (ng/dl)	Borderline (0.02), positive (0.04) after a few hours	> 0.02 ng/dl
Creatinine phosphokinase (ug/L)	493	10-120 ug/L
International normalized ratio	1.51	<1.4
Activated partial thromboplastin time (seconds)	31	30 (seconds)

**Figure 1:**
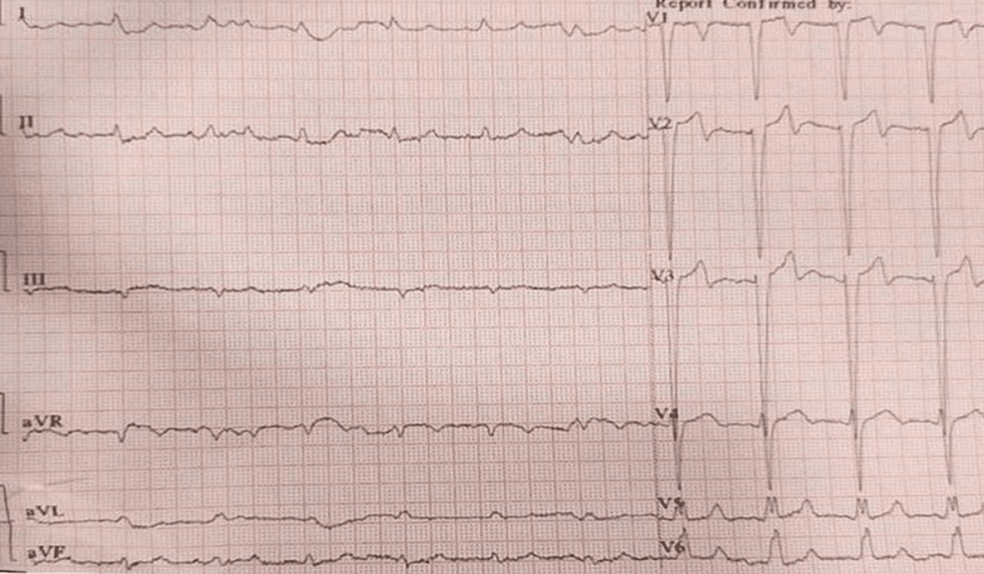
A standard 12-lead ECG showing regular sinus rhythm, heart rate of 88/minute, QRS axis between 00 and +150, normal P wave and a PR interval of 0.14 seconds, normal QRS complex , rsr’ pattern in leads V5 and V6, a QRS interval of 0.12 seconds (intraventricular conduction defect), a QT interval of 40 seconds, T-wave biphasic in V1-V3 and flat to upright in V4, ST segment coving in leads V1 to V4, ST-segment depression in I and aVL, and poor R wave progression.

Her initial ECG showed probable anterolateral wall ST-elevated myocardial infarction (Figure 1).

**Figure 2:**
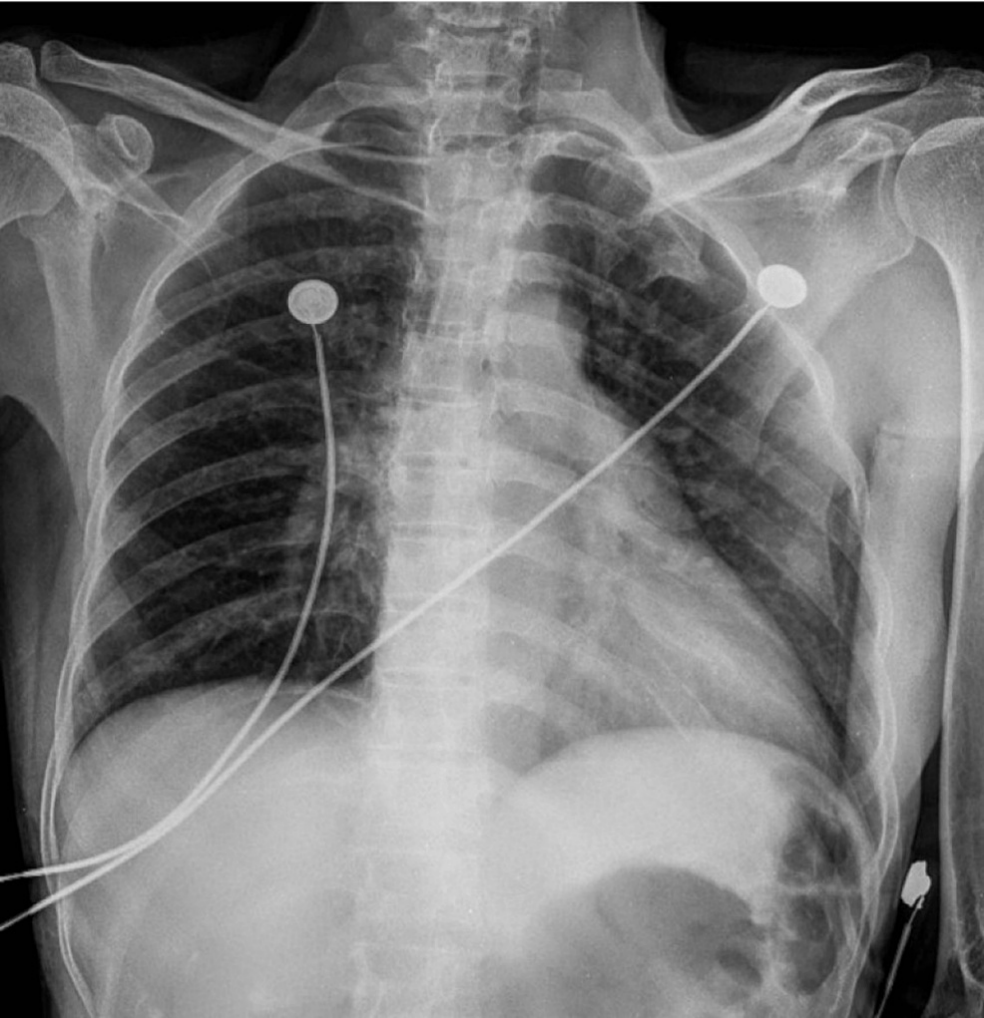
Normal chest X-ray anteroposterior (AP) view. There is no evidence of pulmonary edema.

Her chest X-ray anteroposterior (AP) view showed no abnormality (Figure *2*).

**Figure 3:**
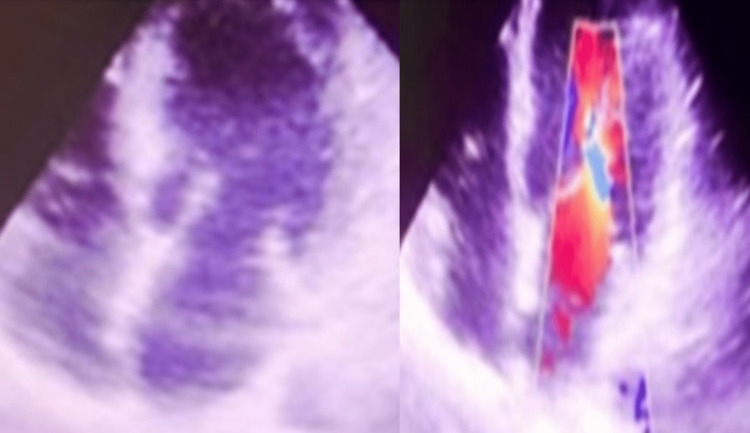
Transthoracic echocardiography apical four-chamber view: (A) anterior, anteroseptal, and septal walls hypokinesia show no evidence of reverse takotsubo; (B) normal valves, trivial mitral regurgitation, and no tricuspid regurgitation were found on color Doppler imaging.

On admission, her transthoracic echocardiogram showed apical wall hypokinesia, valves and inferior vena cava were normal, and there was no evidence of features of reverse takotsubo cardiomyopathy (Figure 3 and Video 1). 

**Video 1:** Anterior, anteroseptal, septal walls hypokinesia, left ventricular ejection fraction of 40%, trivial mitral regurgitation, diastolic function normal (E > A), no tricuspid regurgitation, no pulmonary artery hypertension, and normal inferior vena cava.

**Figure 4:**
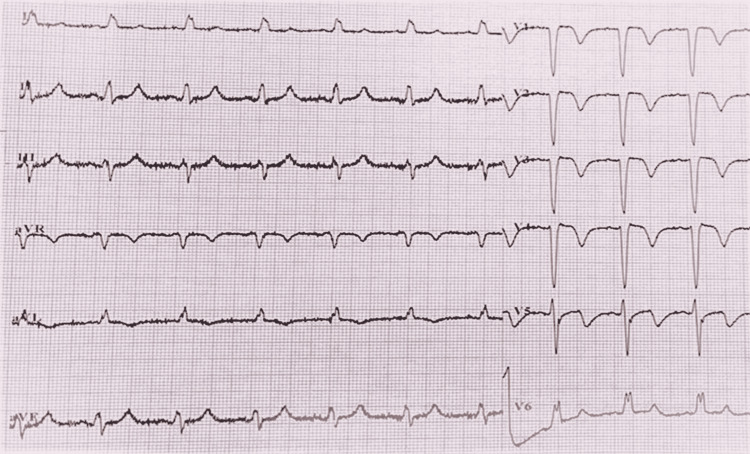
A standard 12-lead ECG showing regular sinus rhythm, heart rate of 75/minute, QRS axis 00, normal P wave and PR interval, slurred R in leads I, aVL, V5, and V6, normal QT interval, elevated S-T segment in V1-V5, deep T-wave inversions in aVL and V1-V4, and biphasic but more negative in V5; findings are suggestive of progressive ischemic changes on Day 2 with intraventricular conduction defect.

**Figure 5:**
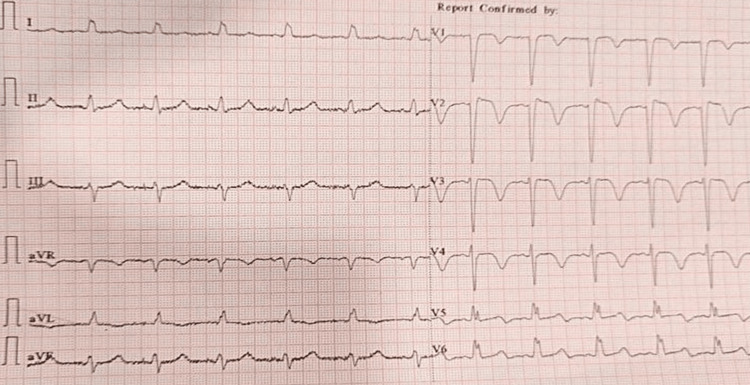
A standard 12-lead ECG showing regular sinus rhythm, heart rate of 75/minute, normal P wave and PR interval, slurred R in leads I, aVL, and V5-V6, prolonged QTc interval, elevated ST segment in V1-V4, T-wave inversions in aVL and V1-V4, and biphasic in V5-V6; results are suggestive of progressive ischemic changes with intraventricular conduction defect on Day 3.

The patient was managed through a two-centimeter diameter ring block with a 2% xylocaine solution (without adrenaline) around the sting site. She was given an injection of Tetanus toxoid 2 ml intramuscular, hydrocortisone 100 mg intravenous, and chlorpheniramine. Her mean arterial pressure (MAP) and urine output were strictly monitored, and her MAP was kept above 65 mmHg. We did not give her scorpion antivenom due to its unavailability. Her serial ECG on Day 2 showed ST-segment elevation in V1-V5, deep T-wave inversions in leads aVL, V1-V4, and biphasic in V5, while on Day 3, ST-segment elevation in V1-V4, T-wave inversions in leads aVL, V1-V4, and biphasic in V5 and V6 were observed, suggestive of progressive ischemic changes (Figures 4 and 5).

We repeated her echocardiography on Day two; the findings are shown in Video 2.

**Video 2:** Anterior, anteroseptal, septal walls hypokinesia, left ventricular ejection fraction: 35%, trivial mitral regurgitation, diastolic function normal (E > A), no tricuspid regurgitation, no pulmonary artery hypertension, and normal inferior vena cava.

We planned to perform coronary angiogram on her to rule out obstructive coronary artery disease, but it could not be performed as the patient did not give consent to the procedure. A coronary computed tomography angiography could not be performed due to its unavailability at our rural hospital. The patient was kept under observation. Upon the resolution of symptoms, she was discharged and followed up on an outpatient department basis.

## Discussion

The scorpion venom is a mixture of water-soluble toxins such as cardiotoxin, neurotoxin, histamine, phospholipases, nephrotoxin, phosphodiesterases, hemolysins, hyaluronidases, and other antigenic compounds [2,4]. Both type I (associated with plaque vulnerability) and type II (associated with a demand-supply mismatch) acute coronary events may occur as a result of scorpion sting. Scorpion stings cause vasospasm of coronary arteries and induce platelet aggregation and facilitate intraarterial thrombosis by acting on coronary endothelium cells. The scorpion venom directly affects the myocardium by reducing Na-K-ATPase, which leads to toxic myocarditis. The toxin also induces the release of adrenaline and noradrenaline from the adrenals, ganglia, and neurons, leading to adrenergic myocarditis. It has direct chronotropic and inotropic effects, which result in increased myocardial oxygen demand in an already compromised myocardium [4]. Scorpion toxin also inhibits the angiotensin-converting enzyme that results in the decreased breakdown of bradykinin, leading to the development of pulmonary edema [2].

Clinical manifestations following a scorpion sting include vomiting, sweating, salivation, priapism, cold extremities, tachycardia or bradycardia, hypertension or hypotension, cardiac arrhythmias, mydriasis, pulmonary edema (autonomic storm), pancreatitis, and neurological manifestations [3,5]. Manifestations following an Indian red scorpion sting vary with the time lapsed from the incident. In Bawaskar and Bawaskar [6], within 1-10 hours, patients presented with hypertension, which was most probably due to sympathetic nervous system stimulation and increased catecholamine release; within the first 24 hours, they presented with tachycardia due to the above reason and there was a direct venomous effect on the cardiac muscles; within 6-24 hours, they presented with acute pulmonary edema.

Scorpion-related cardiomyopathy is a reversible condition characterized by marked alteration in the performance of both ventricles. Initially, there is a vascular phase, which is characterized by vasoconstriction due to catecholamine release, an acute increase in left ventricular (LV) afterload, and systemic hypertension. Hypertension impairs LV emptying and leads to an increase in LV-filling pressure. The second phase is the myocardial phase, characterized by myocardial stunning (decreased LV contractility), leading to low cardiac output and shock stage. In this phase, an alteration in right ventricular function is symmetric to LV function, which is also reversible. This phase may reverse spontaneously or with inotropic treatment [7,8]. Scorpion-related cardiomyopathy may show features of a surge of catecholamines, manifesting in myocardial ischemia due to vasospasms of epicardial coronary and an increase in resistance in coronary arteries. These features resemble takotsubo cardiomyopathy (or stress cardiomyopathy), an emergency medical condition associated with extreme emotional and physical stress. The ECG features suggestive of stress cardiomyopathy, such as ST-segment elevation, low voltage QRS complexes, appearance of transient Q waves, and prolongation of QT interval, were absent in our patient. The classical echocardiographic features of takotsubo cardiomyopathy, such as transient left ventricular mid-segment akinesia and dyskinesia or hypokinesia during systole, with or without apical involvement with hypercontractility of the left ventricle basal segment, were also absent in our patient [8,9].

We also considered the possibility of Kounis syndrome in our patient, but there was no history of allergic reactions such as skin itching, rash (urticaria, erythema, and angioedema), breathlessness and palpitations, nor were hypotension, tachycardia, or bradycardia, cold extremities and wheeze present on examination. Although the absence of skin manifestations is not an exclusion criterion for Kounis syndrome, clinicians should always monitor for severe shock [10,11].

The management of severe scorpion stings comprises fast reduction of raised BP with antagonism of the peripheral action of toxin through alpha-receptor blockers such as prazosin or sublingual nifedipine. Early admission to the hospital is recommended for severe human scorpion stings.

Benzodiazepines are useful as supportive care to decrease painful paraesthesia, which helps alleviate anxiety, and for treatment of myoclonus, opsoclonus, and excessive motor activity in scorpion envenomation [12-14]. Angiotensin-converting enzyme inhibitors (oral captopril) were found to be safe and effective in scorpion stings leading to cardiovascular manifestations in a retrospective analysis. Oral captopril (6.25-25 mg thrice daily) resolved pulmonary edema by afterload reduction in these patients [15].

The studies by Bawaskar and Bawaskar [6,16] on the treatment of scorpion stings with nifedipine showed that nifedipine, when given alone, did not prevent myocardial injury; however, it is effective when administered along with prazosin by blocking the peripheral action of venom. They also studied the role of sodium nitroprusside in the treatment of scorpion stings with 5-10 mcg/min as the initial dose and the maximum tolerated dose titrated up every five minutes from 5 to 400 mcg/min [6,16].

Prazosin is an effective therapy when administered early for *M. tamulus* stings. It improves the clinical symptoms, morbidity, and mortality due to scorpion envenomation, which depends upon the time duration between the sting and administration of prazosin [6,17]. Dobutamine increases myocardial contractility and cardiac output by beta-1 adrenergic action without causing peripheral vasoconstriction through its agonistic action on beta-2 adrenergic receptors in the peripheral blood vessels (as opposed to noradrenaline and vasopressin). This leads to an improvement in hemodynamic parameters and a reduction in mortality in the case of severe scorpion envenomation. The overall treatment of scorpion envenomation-induced pulmonary edema is similar to treating other causes of cardiogenic pulmonary edema [18].

Scorpion antivenom should be administered intravenously as early as possible for better diffusion. The dosage of antivenom is based on the grade of envenomation, time lapsed between the sting and treatment initiation, progression of symptoms, and the formulations and titer of antivenom. The neutralizing action of scorpion antivenom is seen only on circulating and tissue venom, but it does not affect receptors. Both monovalent and polyvalent antivenom rapidly and effectively reduce scorpion venom symptoms [19].

There are few published cases of myocardial infarction after a scorpion sting in adults [1,4,7,20]. These studies have rural settings and patients who presented within four hours of the sting. In three studies, chest pain, breathlessness, and sweating were the presenting complaints. The clinical characteristics, laboratory reports, treatment, and outcome of four patients, along with the patient described in the current report, are summarized in Table 3.

**Table 3:** Literature review of cases of scorpion sting with presentation as acute myocardial infarction.

Serial number	Observations	Author references				
		Maheshwari et al. [4]	Agrawal et al. [1]	Abroug et al. [7]	Ismail et al. [20]	Current case
1	Time of presentation since the scorpion sting	30 minutes	3-4 hours	3-4 hours	30 minutes	2 hours
2	Presenting complaints	Chest pain, breathlessness, and sweating	Chest pain, vomiting, loose stools, sweating, cough, breathlessness, and palpitations	Chest pain and breathlessness	Nausea, vomiting, dizziness, and profuse sweating	Local pain and profuse sweating
3	ECG changes					
	Day 1	Sinus tachycardia with secondary ST-T changes	Sinus tachycardia with secondary ST-T changes	Normal sinus rhythm	Sinus tachycardia with diffuse ST depressions	Sinus rhythm, ST segment coving in leads V1-V4, ST segment depression in leads I and aVL, and poor R wave progression
	Day 2	Sinus rhythm with T-wave inversions in leads 1, aVL, and V1-V2, ST-segment elevation with concavity upward in leads II, III, aVF, and V5-V6	Sinus rhythm with ST-segment elevation with concavity upward in leads II, III, aVF, and V3-V6, T-wave inversion in leads 1, aVL, and V1 and V2		Spontaneously resolving short runs of ventricular tachycardia	Sinus rhythm, slurred R in I, aVL, V5, and V6, S-T segment elevation in V1-V5, deep T-wave inversions in leads aVL and V1-V4, and biphasic in V5
	Subsequent	After one month: Symmetric T-wave inversions in leads II, III, aVF, and V2-V6	On Day 5: Persistence of Q-wave and T-wave inversions in leads I and aVL		On Day 3: Sinus rhythm with a resolution of ST changes and prolongation of QT interval	On Day 3: S-T segment elevation in V1-V4, T-wave inversions in leads aVL and V1-V4 and biphasic in V5-V6
4	Troponin T	0.06 ng/dl	0.382 ng/dl	-	0.26 ng/dl	
5	Creatinine phosphokinase	45 IU/L	15.46 IU/L	-	52 ng/ml	
6	Echocardiogram					
	Day 1	No RWMA and normal valves		Basal ballooning of the left and right ventricles, an LVEF of 35%	Severe left ventricular global hypokinesia with an LVEF of 35%, and mild mitral regurgitation	Apical wall hypokinesia, an LVEF of 40%, and normal valves and IVC
	Day 2	Hypokinesia of interventricular septum and inferior posterior wall with moderate mitral and tricuspid regurgitation; an LVEF of 28%	Hypokinesia of interventricular septum and inferior posterior wall with moderate mitral and tricuspid regurgitation; an LVEF of 32%.			Apical wall hypokinesia with LVEF- 35%, valves, and IVC normal
	Subsequent	After one month: No RWMA with well-preserved LVEF	After five days: No RWMA, Mild mitral and tricuspid regurgitation; an LVEF of 56%			After one month: No RWMA and normal valves and IVC, and an LVEF of 51%
6	Chest X-ray	Pulmonary edema	Pulmonary edema	Pulmonary edema	Pulmonary edema	Normal
7	Patients received prazosin (Yes/No)	No	No	No	Yes	Yes
8	Patients received anti-scorpion venom (Yes/No)	No	No	No	Yes	No
9	Patients received a cardiac intervention	Antiplatelets, low-dose beta blockers, coronary angiogram suggestive of normal coronary vessels with positive ergonovine, and acetylcholine challenge test	Intravenous dopamine, dobutamine, noradrenaline, frusemide, corticosteroids, oral aspirin, nitrates, and non-invasive oxygen therapy in the form of continuous positive airway pressure	No	Scorpion antivenom, corticosteroids, dopamine, noradrenaline, antibiotics, and mechanical ventilation	Antiplatelets (oral aspirin), corticosteroids, and non-invasive oxygen therapy
10	Outcome	Discharged and had no complications during the follow-up period	Discharged	Discharged	Discharged and had no complications during the follow-up period	Discharged and had no complications during the follow-up period

We are reporting a case in adult patients; hence, pediatric cases have not been included in the above table.

## Conclusions

Pain at the sting site and sweating are considered the main symptoms of a scorpion sting. However, as this case illustrates, severe cardiovascular manifestations, including acute myocardial infarction, are also observed in scorpion sting patients experiencing chest pain and breathlessness. Failure to recognize and treat this early can be fatal. Thus, prompt treatment after immediate hospitalization with alpha-1 receptor blocker such as prazosin is essential to resolve clinical symptoms and prevent complications in scorpion-stung patients.
